# Supplementary material for: Ultrasensitive one-pot detection of monkeypox virus with RPA and CRISPR in a sucrose-aided multiphase aqueous system
Source: Microbiol Spectr. 2023 Dec 11;12(1):e02267-23. doi: 10.1128/spectrum.02267-23 (PMC10782985; doi:10.1128/spectrum.02267-23)
Supplement: Supplemental material — Fig. S1 and S2; Table S1. [file spectrum.02267-23-s0001.pdf]

Ultrasensitive one-pot detection of monkeypox virus with RPA and CRISPR in a sucrose-aided multiphase aqueous system

Yue Wang [a, 1], Yixin Tang [a, 1], Yukang Chen [a], Guangxi Yu [a], Xue Zhang [a], Lihong Yang [a], Chenjie Zhao [a], Pei Wang [b, *], Song Gao [a, *]

[a] *Jiangsu Key Laboratory of Marine Biological Resources and Environment, Co-Innovation Center of Jiangsu Marine Bio-industry Technology, Jiangsu Key Laboratory of Marine Pharmaceutical Compound Screening, Jiangsu Ocean University, Lianyungang 222005, China*

[b] *School of Food Science and Pharmaceutical Engineering, Nanjing Normal University, Nanjing 210023, China*

[1] Y. W. and Y. T. contributed equally to this work.

[*] Corresponding authors.

E-mail addresses: 90830@njnu.edu.cn (P. Wang); gaos@jou.edu.cn (S. Gao)

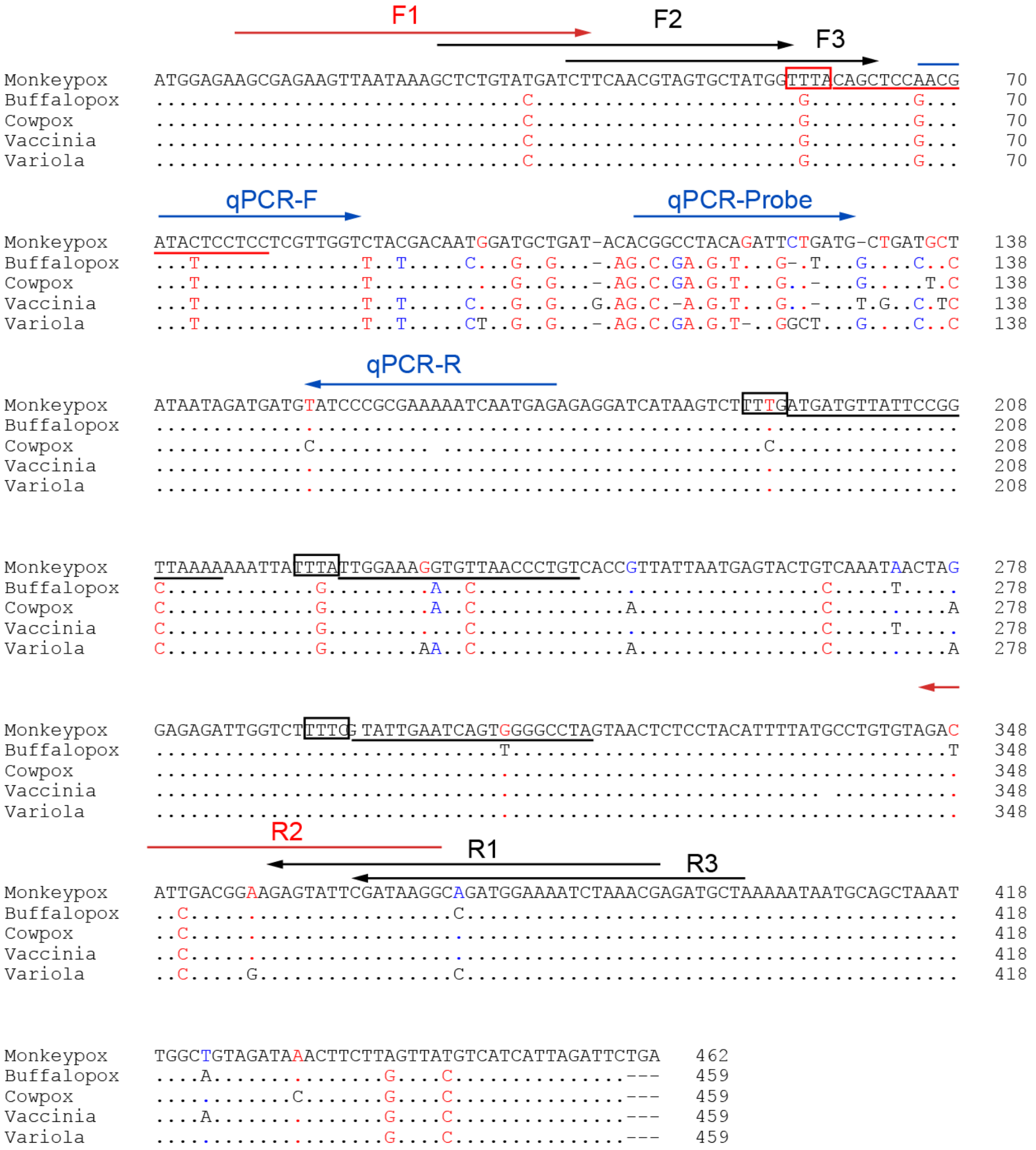

Figure S1. Sequence alignment of the homologous regions of the monkeypox virus F3L gene with the F3L-homologous genes from other human-related orthopoxviruses. The positions corresponding to the RPA primers (forward: F1-3; reverse: R1-3) are indicated by horizontal arrows. The PAM motifs are boxed and corresponding sequences for the crRNAs are underlined. The positions corresponding to qPCR primers and probe of a standard monkeypox test (The Codes for Examination of Monkeypox at Entry-exit Port of China, SN/T 2097-2008) are indicated by blue horizontal arrows for readers' reference.

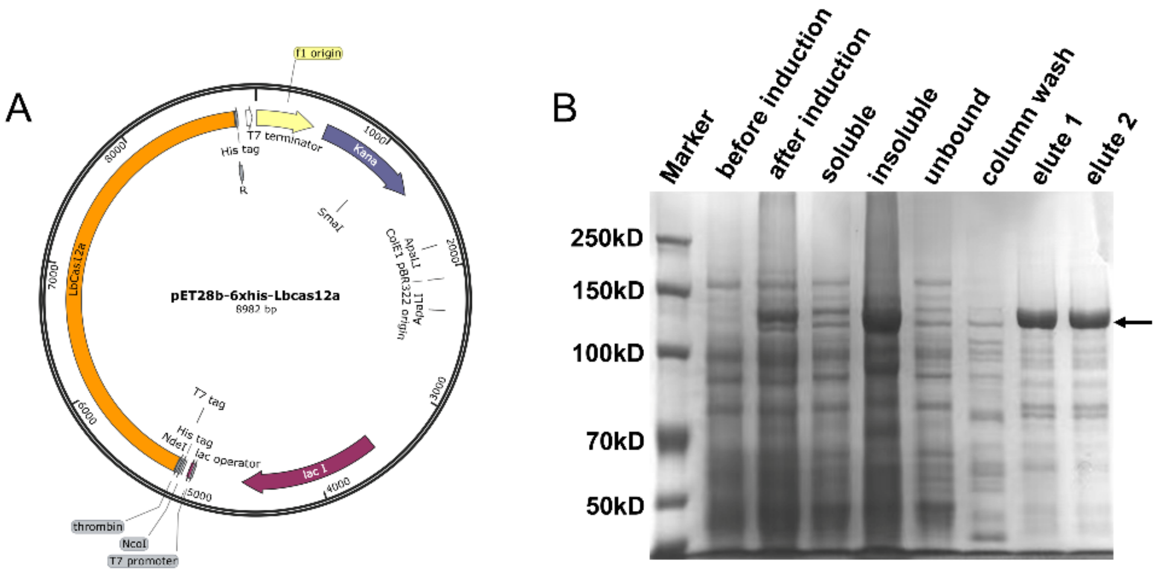

Figure S2. Expression and purification of Cas12a. (A) Map of the recombinant plasmid for the overexpression of Cas12a. The key expression elements are indicated. (B) SDS-PAGE analysis of the overexpression and purification of Cas12a by Ni-affinity chromatography. The band position corresponding to the recombinant Cas12a is indicated by an arrow.

TABLE S1 Recent studies on molecular detection of monkeypox

No.	Detection method	Target gene(s)	Sensitivity	Time	Reference*
1	Cas12a	F3L and N3R	10^9 copies	10 min	(1)
2	LAMP	F3L and A27	20 copies	60 min	(2)
3	RPA-Cas12a one-step	B7R	15 copies	35 min	(3)
4	Real-time PCR	E9L and B6R	10 copies	35 min	(4)
5	Real-time RPA	G2R	10^0 copies	41 min	(5)
6	RAA-LFS	G2R	10^0 copies	35 min	(5)
7	RPA-Cas12a two-step	G2R	10^0 copies	62 min	(5)
8	LAMP-Cas12b two-step	D14L and ATI	10 copies	42 min	(6)

*** References related to this table:**

1. Sui Y, Xu Q, Liu M, Zuo K, Liu X, Liu J. 2022. CRISPR-Cas12a-based detection of monkey pox virus. J Infect 85:702-769. https://doi.org/10.1016/j.jinf.2022.08.043.
2. Feng J, Xue G, Cui X, Du B, Feng Y, Cui J, Zhao H, Gan L, Fan Z, Fu T, Xu Z, Du S, Z hou Y, Zhang R, Fu H, Tian Z, Zhang Q, Yan C, Yuan J. 2022. Development of a loop-medi ated isothermal amplification method for rapid and visual detection of monkeypox virus. Micro biol Spectr 10:e0271422. https://doi.org/10.1128/spectrum.02714-22.
3. Chen Q, Gul I, Liu C, Lei Z, Li X, Raheem MA, He Q, Haihui Z, Leeansyah E, Zhang CY, Pandey V, Du K, Qin P. 2023. CRISPR-Cas12-based field-deployable system for rapid detectio n of synthetic DNA sequence of the monkeypox virus genome. J Med Virol 95:e28385. https://doi.org/10.1002/jmv.28385.
4. Li Y, Olson VA, Laue T, Laker MT, Damon IK. 2006. Detection of monkeypox virus with rea l-time PCR assays. J Clin Virol 36:194-203. https://doi.org/10.1016/j.jcv.2006.03.012.
5. Mao L, Ying J, Selekon B, Gonofio E, Wang X, Nakoune E, Wong G, Berthet N. 2022. Deve lopment and characterization of recombinase-based isothermal amplification assays (RPA/RAA) f or the rapid detection of monkeypox virus. Viruses 14:2112. https://doi.org/10.3390/v14102112.
6. Chen X, Yuan W, Yang X, Shi Y, Zeng X, Huang J, Wang Y, Li S. 2023. Ultrasensitive and specific identification of monkeypox virus Congo Basin and West African Strains using a CRIS PR/Cas12b-based platform. Microbiol Spectr 11:e0403522. https://doi.org/10.1128/spectrum.04035-22.
